# Anxiety-related behaviors without observation of generalized pain in a mouse model of endometriosis

**DOI:** 10.3389/fnbeh.2023.1118598

**Published:** 2023-02-09

**Authors:** Paulina Nunez-Badinez, Alexis Laux-Biehlmann, Michael D. Hayward, Olesia Buiakova, Thomas M. Zollner, Jens Nagel

**Affiliations:** ^1^Exploratory Pathobiology, Research and Early Development, Research and Development, Bayer AG, Wuppertal, Germany; ^2^Taconic Biosciences, Cranbury, NJ, United States; ^3^Endocrinology, Metabolism and Reproductive Health, Research and Early Development, Research and Development, Bayer AG, Berlin, Germany

**Keywords:** preclinical research, endometriosis, anxiety, *in vivo*, rodent, translational research

## Abstract

Endometriosis is a chronic, hormone-dependent, inflammatory disease, characterized by the presence and growth of endometrial tissue outside the uterine cavity. It is associated with moderate to severe pelvic and abdominal pain symptoms, subfertility and a marked reduction in health-related quality of life. Furthermore, relevant co-morbidities with affective disorders like depression or anxiety have been described. These conditions have a worsening effect on pain perception in patients and might explain the negative impact on quality of life observed in those suffering from endometriosis-associated pain. Whereas several studies using rodent models of endometriosis focused on biological and histopathological similarities with the human situation, the behavioral characterization of these models was never performed. This study investigated the anxiety-related behaviors in a syngeneic model of endometriosis. Using elevated plus maze and the novel environment induced feeding suppression assays we observed the presence of anxiety-related behaviors in endometriosis-induced mice. In contrast, locomotion or generalized pain did not differ between groups. These results indicate that the presence of endometriosis lesions in the abdominal cavity could, similarly to patients, induce profound psychopathological changes/impairments in mice. These readouts might provide additional tools for preclinical identification of mechanisms relevant for development of endometriosis-related symptoms.

## Introduction

Endometriosis is characterized by the presence of uterine stroma and glands outside the uterine cavity, predominantly on the pelvic peritoneum and ovaries. It affects approximately 5–10% of women of reproductive age and represent a significant disease burden (Shah and Jagani, 2018; Simitsidellis et al., 2018; Zondervan et al., 2018). While pelvic and abdominal pain symptoms (e.g., chronic pelvic pain, dysmenorrhea, dyspareunia) and subfertility are the most common clinical signs of endometriosis (Mechsner, 2016), many relevant co-morbidities with affective disorders like depression or anxiety are described and have severe impact on quality of life of patients (Brandes, 2007; Facchin et al., 2015, 2017). The association between endometriosis and mental illnesses has been reported by several studies and confirms a significant incidence of anxiety, depression, and psychopathological symptoms among women with endometriosis [for review, (Laganà et al., 2017)].

Various animal models have been established for the identification and development of drugs for the treatment of affective disorders. In such models, a behavioral characterization of complex cognitive and//or emotional processes is required. For example, the forced swimming test and the sucrose preference test have been widely used to evaluate depression-like behaviors, and the open field test, the novelty suppressed feeding, and the elevated plus maze (EPM) test, have been used to evaluate anxiety-related behaviors. Outcomes such as behavioral despair, immobility responses, anhedonia; as well as locomotion, thigmotaxis, latency to approach food and/or food intake, and time spent in open/illuminated vs. closed/dark areas are quantified. The antidepressant and anxiolytic activities of potential therapeutic agents can be evaluated by the degree of reduction of such behavioral responses upon agent administration (Belovicova et al., 2017).

In endometriosis research it is crucial to understand the complex pathophysiology of endometriosis as well as the identification and development of new therapeutic options [for review: (Simitsidellis et al., 2018)]. The availability of rodent models, their validity for translational research (e.g., construct, face, and predictive validity) and the quality of their characterization are of evident importance. A recent literature survey of preclinical models for endometriosis shown that the most common outcome measures are biological and histopathological evaluations of the lesions and identification of inflammatory markers. In strong contrast, the behavioral outcomes related with pain have been rarely studied and are fundamental to improve the validity of these models for the effective development of new therapies for endometriosis (Nunez-Badinez et al., 2021). In the present study, we aim to characterize the behavioral phenotype resulting from surgery-induced endometriosis in mice, using established anxiety and pain assays.

## Methods

### Animals

*In vivo* experiments were performed by Taconic Biosciences, a contract research organization located in the USA providing animal facilities accredited by the association for Assessment and Accreditation of Laboratory Animal Care (AAALAC) which follows the guidelines of the International Association for the Study of Pain for use of Animals in Research. The facilities were reviewed and approved by the American Preclinical Services Institutional Animal Care and Use Committee (IACUC) prior to study initiation.

Female adult C57BL/6 mice (8–10 weeks; Taconic) were acclimatized for 1–3 weeks and maintained group housed (3–4 animals/cage) in a 12h:12h light:dark cycle with access to food and water *ad libitum*. Animals were randomly allocated to treatment groups on the day of surgery from a stock and were uniquely identified by ear tags. A total of 48 animals were included, with an *n*/group = 24. Two studies were performed, and per study the *n*/group was 12. One sham mouse was afterwards identified as an outlier and excluded from the study.

### Surgically induced endometriosis

**FIGURE 1:**
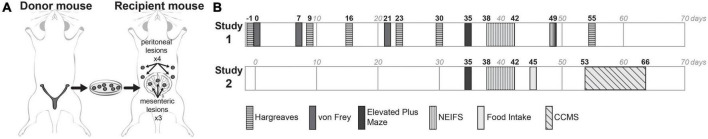
Experimental plan. **(A)** For the induction of endometriosis in mice, the syngeneic transplantation model was performed surgically implanting seven biopsies into recipient mice: three biopsies from donor mouse were attached on the mesentery of the small intestine and four on the abdominal wall of the interior of the abdomen. **(B)** Two independent studies were executed, with evaluation of different endpoints. In the first study (study 1) mechanical and thermal pain-related behaviors were assessed with the von Frey test and Hargreaves test at different timepoints, and anxiety-related behaviors were performed by the EPM and NEIFS assays at days 35 and 38–42 after surgery. In the second study only anxiety-related behaviors were measured at the same timepoints. Additionally, monitoring of locomotive activity by the CCMS assay was performed at days 53–66 after surgery.

Endometriosis was mimicked in mice by syngeneic transplantation of uterine tissue on the intestinal mesentery and the peritoneal wall (Figure 1A). In brief, animals were deeply anaesthetized with continuous inhalation of isoflurane 5% (Butler Schein-Aerrane; Baxter Healthcare Corp. #NDC 10019-773-60) and uterine horns from donor mice in estrus were removed, opened longitudinally in warm HBSS solution, and cut into 2-mm fragments using a dermal biopsy punch. In recipient mice, the abdominal cavity was longitudinally opened, and three biopsies were attached on the mesentery of the small intestine and four were attached to the abdominal wall (2 on each side) of the interior of the abdomen using 6–0 proline sutures (J&J, Ethicon). The abdominal wall and the skin openings were subsequently closed using sutures. Sham mice were subjected the same procedure, but in place of suturing uterine tissue, empty sutures were realized.

### Study design

Two studies were performed (Figure 1B), with a group size of 12/group/study. Both studies assessed anxiety-like behaviors by the EPM test (Figure 1B, black rectangles) and the Novel Environment-Induced Feeding Suppression (NEIFS) assay (rectangles with vertical lines). In the first study, additional assessments were performed to determine if endometriosis had any effects on generalized mechanical or thermal pain, evaluated by the von Frey and the Hargreaves test (rectangles with horizontal lines). In the second study, to determine if endometriosis had any effects on locomotor activity, the Comprehensive Cage Monitoring System (CCMS) was recorded (rectangle with diagonal lines). Food intake of the animals was measured at post-surgical days 45 (study 2) and 49 (study 1, solid gray rectangles). Behavioral tests were done during the light phase of the cycle in standard lightning conditions, at the same time of the day, and experimenters that performed these tests were blinded to the groups. On testing days, mice were acclimated to the room 30 min prior to testing.

### Elevated plus maze (EPM)

The EPM test protocol was realized as previously described (Walf and Frye, 2007; Komada et al., 2008). Briefly, the maze consisted of two opposing open arms (H:0.5 × L:25 × W:5 cm each) and two opposed closed arms (H:16 × L:25 × W:5 cm each), with an elevation of approximately 50 cm above floor. Animals were placed at the center of the maze facing a closed arm and recorded for 5 min. Time spent in the open and closed arms of the EPM and the number of entries into the arms was determined using Videotrack (Viewpoint Lifesciences, Montreal, Canada) software. The number of entries into the open arms, normalized to the total number of entries was determined as an internal control for locomotor activity.

### Novel Environment-Induced Feeding Suppression (NEIFS)

A Petri dish containing crushed Graham crackers was placed in the farthest corner from each mouse in its home cage for three consecutive days (days 1, 2, and 3) and day 5. On day 4, mice were placed in a clean cage (novel environment) and the same procedure was realized. The latencies to approach and to consume (eat, not pick up) the crackers were recorded immediately following the placement of the dish into home cage. Animals were not fasted prior this test. A cut-off time of 10 min was enforced if the mouse failed to consume the food. The remaining crackers and Petri dish were discarded at the end of the experiment.

### Thermal pain thresholds detection

On the day of testing, the detection of thermal pain thresholds (TPT) was performed in Hargreaves’ chambers (IITC Life Science, Woodland Hills, CA, USA) as previously described (Deuis and Vetter, 2016). Briefly, the radiant heat beam was focused on the plantar surface of each hind paw and the time taken to withdraw the paw was recorded with a cut off time of 20 s to prevent tissue damage. The mean time to withdrawal was determined from the average of 2 tests, separated by 5 min.

### Mechanical pain thresholds detection

50% mechanical threshold was determined for each hind paw by Dixon’s up-and-down method (Dixon, 1991) using calibrated von Frey hairs (Stoelting, Wood Dale, IL; USA; 0.16, 0.40, 0.60, 1.00, 1.40, 2.00, and- 4.00 g). The measurement started with the 1 g fiber applied perpendicular to the plantar surface of the hind paw with enough force to bend the fiber, held for 2 s and the response was recorded. A positive response was defined as shaking, licking, biting, or withdrawing the paw during or up to 2–3 s after stimulation. A negative response was defined as a lack of the described behaviors. An interval of minimum 30 s was given between two consecutive readings from the same mouse and at least 1 min for the same paw. If the fiber resulted in a positive response, the next lower fiber was used. If the fiber resulted in a negative response, the next higher fiber was used. The first positive change was defined as the first positive response. The next four responses following the positive change were recorded and used to calculate the 50% mechanical threshold as follows:

50% mechanical threshold (g) = 10 ^Xf + kd^, where:

X_f_ = the force exerted by the final von Frey hair used (log units),

k = tabular value for the pattern of positive/negative responses (Chaplan et al., 1994),

d = mean difference (log units) between each pair of stimulus fibers.

### Food intake

Food intake measurements were performed over a 4 h period at the beginning of the dark phase of the photoperiod. The mice were single-housed and given enough food to fill the food container. At the end of the period, the remaining food was recovered and weighed.

### Comprehensive Cage Monitoring System (CCMS)

The CCMS (Columbus Instruments, Columbus, OH, USA) was used according to manufacturer’s protocol and as previously described (Tsao et al., 2008; Steen et al., 2013). Briefly, CO_2_ production, O_2_ consumption, water intake and locomotor activity (vertical and horizontal) were monitored for 2 days. Activity endpoints were recorded as the cumulated values counted by the detectors during light phase. Group size was *n* = 8.

### Statistical analyses

Data is shown as mean ± standard error of the mean (SEM). Statistical significance was set at *p* < 0.05. For statistical analyses at a single time point, such as in the EPM test as well as individual food intake, the unpaired t-test was used. For analysis at multiple time points, such as the NEIFS test, the MPT, TPT, and the CCMS assay, two-way ANOVA tests were performed. *Post hoc* pairwise comparisons were made using least significant difference (LSD). To compare changes between groups, a multiple *t*-test corrected for multiple comparisons with the Holm-Sidak method was used. A “trend” is defined as 0.1 > *p* > 0.05. The levels of significance (*p* < 0.001, < 0.01, and < 0.05) for comparisons within endometriosis-induced mice (EIM) at different timepoints and between EIM and sham groups are shown as ^***^, ^**^, and *, and within sham mice as °°°, °°, and °. Outliers were defined by a value being two times the standard deviation of the mean of the experimental group.

## Results

### Pain-related behaviors

#### Hind paw mechanical pain thresholds of EIM remained unchanged

**FIGURE 2:**
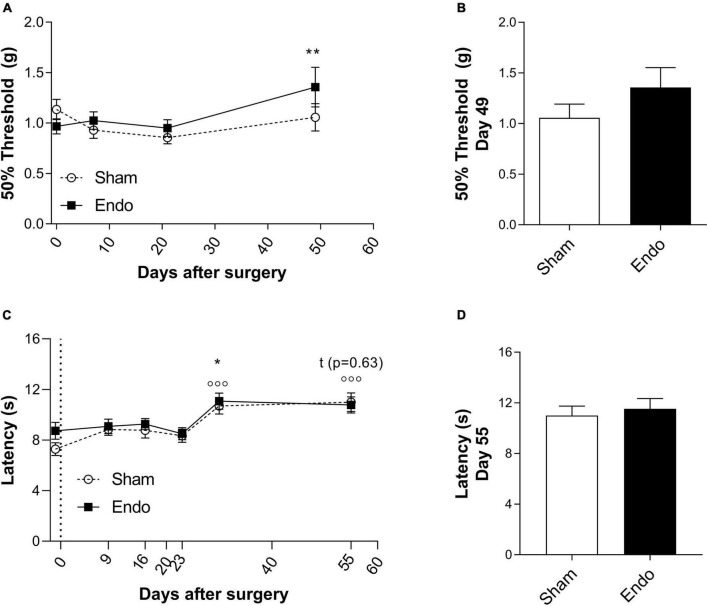
Pain-related behavior in endometriosis-induced mice. MPT **(A,B)** and TPT **(C,D)** measured by the von Frey and the Hargreaves test in sham (*n* = 23) and EIM (*n* = 24). **(A)** Time course of the MPT (expressed in means of 50% force to react to the mechanical probe) of sham and EIM along 49 days, at days 0 (baseline), 7, 21, and 49 after surgery. **(B)** MPT between EIM and sham mice at day 49 after surgery. **(C)** Time course of the TPT (expressed in means of time to react to the heat stimulus) of sham and EIM along 55 days, measured on days: −1, 9, 16, 23, 30, and 55 after surgery. **(D)** TPT between EIM and sham mice at day 55 after surgery, no significant differences are observed. Data shown as mean ± SEM. * = *p* < 0.05.

To analyze if endometrial implants would influence mechanical pain thresholds (MPT), the von Frey test was assessed at days 0, 7, 21, and 49 post-surgery (Figures 2A, B). After model induction, no differences in MPT between groups were observed. When compared within groups to their respective baseline MPTs, only at day 49 there is a significant increase in MPT in EIM (Figure 2A, 2-way ANOVA with multiple comparisons, *p* < 0.01), indicating an increase in pain tolerance. No differences in MPTs between groups were found at day 49 (Figure 2B, unpaired *t*-test *p* = 0.22), indicating that endometrial implants do not alter the MPT at the hind paws of mice.

#### EIM did not alter hind paw thermal pain thresholds

To test changes on TPT in EIM, Hargreaves test was executed at days: –1, 9, 16, 23, 30, and 55 post-surgery (Figures 2C, D). In both groups, compared to their respective baselines, no significant changes on TPT on days 9, 16, and 23 were observed (Figure 2C, 2-way ANOVA with multiple comparisons, Sham *p* = 0.26, 0.31, and 0.68; EIM *p* = 1.00, 0.98, and 1.00, respectively). On day 30, both groups displayed a significant increase on the withdrawal latency compared to baseline (Figure 2C, 2-way ANOVA with multiple comparisons, Sham *p* < 0.001, EIM *p* < 0.05). No differences between groups were found in TPT at day 55 (Figure 2D, unpaired *t*-test *p* = 0.63). Altogether, endometrial implants did not affect the TPT in the hind paws, and if at all, repeated evaluations might even increase heat tolerance.

### Anxiety-related behaviors

#### EIM are less prone to expose themselves in an open environment

**FIGURE 3:**
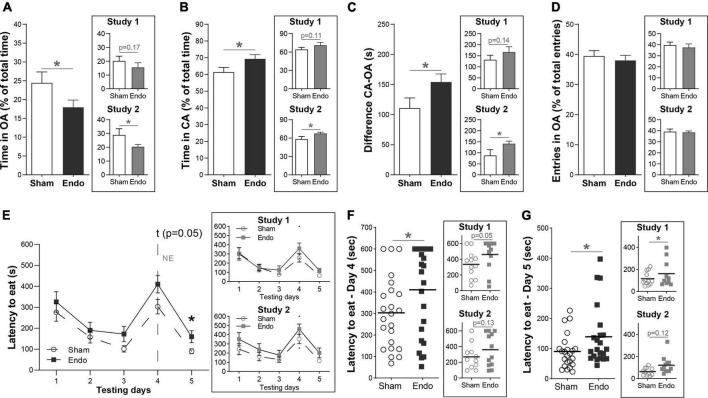
Anxiety-related behavior in endometriosis-induced mice. **(A–D)** Measurements of the EPM assay in means of time spent and number of entries between the open and closed arms of the instrument. **(A)** Comparison of the time spent in the open arm (OA) of the EPM between EIM and sham mice. **(B)** Comparison of the time spent in the closed arm (CA) of the EPM between EIM and sham mice, shown as percentage from the total time of the assay. **(C)** Difference between time spent in the CA and the OA of the instrument. **(D)** Number of entries in the OA, shown as percentage from the total number of entries. **(E–G)** Measurements of the NEFS assay in means of latency to approach food in a novel, open environment. **(E)** Mean latency to approach food among testing days between EIM and sham mice. On testing day 4, food and mice were in an open, novel environment. **(F,G)** Comparison of the latency to approach food on testing day 4 **(F)** and testing day 5 **(G)** between EIM and sham mice. Dots represent the value of each mouse. Horizontal bars: mean value within each group. The main panels show data pooled from two independent studies (*n* sham = 23, *n* EIM = 24), each of them shown separately in insets (study 1: *n* sham = 12, *n* EIM = 12; study 2: *n* sham = 11, *n* EIM = 12). Data shown as mean ± SEM. * = *p* < 0.05.

To evaluate whether EIM present altered anxiety levels, we first assessed time spent in the open and closed arms of the EPM assay 35 days post-surgery (Figures 3A–D). EIM spent significantly less time in the open arms, compared to sham (Figure 3A, EIM 53.50 ± 10.95 s, 17.94 ± 1.93%; Sham 73.27 ± 10.95 s, 24.43 ± 2.90%; *p* < 0.05), and spent significantly more time in the closed arms (Figure 3B, EIM 208.20 ± 10.95 s, 69.33 ± 2.56% Sham 184.10 ± 10.95 s, 61.37 ± 2.97%; *p* < 0.05). Consequently, the difference between time spent in the closed and the open arms was also increased (Figure 3C, EIM 154.13 ± 13.33 s; Sham 110.79 ± 16.89 s; *p* < 0.05). No differences between groups were found in the number of entries to the open arms, indicating that the locomotor activity of these mice was not affected by surgery (Figure 3D, EIM 38.00 ± 1.68%; Sham 39.44 ± 1.78%; *p* = 0.56). In summary, endometriotic implants reduced the time spent in the open arms of the EPM assay.

#### EIM show increased latencies to eat in a novel environment

During post-surgical days 38–42 we performed the NEIFS assay, measuring the latency to consume palatable food in both their home cage and in a novel, open environment. Consumption latencies differed significantly across experimental days (mixed-effects analysis, effect of days *p* < 0.001). During the first 3 days, the latency to approach food decreased constantly in both groups similarly (Figure 3E, *p* = 0.44, 0.50, and 0.08 for each day). On day 4, the novel environment day, EIM displayed a trend toward a longer consumption latency compared to sham (Figure 3E, multiple comparisons with LSD *post hoc* analysis: *p* = 0.05). On day 5, when food was offered again in their own home cages, EIM displayed a longer latency to approach food compared to sham (Figure 3E, multiple comparisons with LSD *post hoc* analysis; *p* < 0.05). Within groups, the consumption latency on day 4 was significantly longer compared to day 3 (Dunnet’s multiple comparisons test, *p* < 0.001) and to day 5 (*p* < 0.001). Between groups, the consumption latency on the novel environment day was significantly longer in EIM compared to sham (Figure 3F, EIM: 410 ± 42 s, Sham: 303 ± 34 s, unpaired *t*-test, *p* < 0.05) and this increased latency is maintained at the following day, when animals were fed in their original home cages (Figure 3G, EIM: 139 ± 21 s, Sham: 90 ± 12 s, unpaired *t*-test, *p* < 0.05).

In summary, EIM displayed an increased latency to approach a palatable food, validating the effect of the novel environment in this assay, that is, a neophobic response. Despite being a mild effect, it is sustained even when animals come back to their own home cages, suggesting the existence of a second anxiety-related behavior.

#### Monitoring locomotion and metabolic parameters by CCMS

Continuous monitoring of locomotor activity was tracked during a 2-week period (post-surgical days 53–66) using CCMS. No specific group differences were observed in horizontal nor vertical activity, as well as in metabolic indexes: water drinking, oxygen consumption, CO_2_ production, respiratory exchange ratio (RER), or heat production (Supplementary Figure 1). Therefore, under the conditions tested, EIM do not show alterations in their locomotor or metabolic activity, compared to sham mice.

## Discussion

Endometriosis, characterized by the presence and growth of endometrial tissue outside the uterine cavity, often cause pelvic and abdominal pain in patients, subfertility and a marked reduction in health-related quality of life (Imboden and Mueller, 2018; Zondervan et al., 2018). Moreover, co-morbidities with affective disorders like depression or anxiety have also been described (Laganà et al., 2017). Several studies using endometriosis rodent models are demonstrating biological and histopathological similarities of these models with the human situation, but their behavioral characterization was never performed. To elucidate how the induction of experimental endometriosis might affect pain sensation and anxiety in mice, we performed two studies: In the first study both pain- and anxiety-related behaviors were assessed; and the second study focused on anxiety-related behaviors. Our main findings are that EIM versus sham group do not show significant signs of generalized mechanical- nor thermal-induced pain states; and second, despite this, EIM do display anxiety-related behaviors.

The quality of an animal model for translational research relies on three different criteria: construct-, face-, and predictive validity (Vervliet and Raes, 2013), meaning that (1) the etiology (2) the symptoms/hallmarks observed, and (3) the effect of treatment with standard of care should be, ideally, directly translatable to the original disease. In endometriosis, many theories about its pathogenesis have been proposed (Zondervan et al., 2018) and therefore, the construct validity of the animal models relies on them. Retrograde menstruation is a physiological process in which menstrual tissue flows backwards *via* fallopian tubes and deposits tissue in the intraperitoneal space, providing a source for the development of endometriotic lesions. It is hypothesized that this process contributes to the development of endometriosis. Additionally, factors causing a high endometrial flow (e.g., early menarche, no pregnancies, late menopause) are discussed as risk factors in woman to develop endometriosis. Transplanting endometrial tissue samples onto peritoneum and mesenteric arteries intends to model the consequences of retrograde menstruation and high endometrial flow. In the syngeneic transplantation model, this is achieved by mostly two means: suture or intraperitoneal inoculation of endometrial biopsies from donor into recipient mice. The latter has been proposed as having a higher construct validity as the intervention is less invasive and mimics retrograde menstruation (Nunez-Badinez et al., 2021). Nevertheless, both methods induce endometriotic-like lesions with dense vascularization, adhesions and secretion of cytokines in the implanted areas and surroundings (Somigliana et al., 1999; Bordignon et al., 2013; Simitsidellis et al., 2018). Furthermore, antiangiogenic therapy eradicates the lesions (Dabrosin et al., 2002), supporting predictive validity (Edwards et al., 2013). However, other important symptoms/hallmarks of endometriosis, like anxiety or depression (Laganà et al., 2017) have not yet been evaluated; therefore, the face validity is not well characterized. We will discuss studies that have measured pain and anxiety in syngeneic models of endometriosis and compare their results with ours.

The incidence of anxiety and depression traits in endometriosis patients has been reported in many clinical studies (Sepulcri and Amaral, 2009; Facchin et al., 2015, 2017; Friedl et al., 2015; Laganà et al., 2015; Chen et al., 2016) and reviewed (Pope et al., 2015; Laganà et al., 2017). Chen et al. (2016) reported that endometriosis patients have an increased risk of developing major depression, any depressive disorder, and anxiety disorders in later life compared to age and sex-matched controls. Additionally, depressive and anxiety disorders accounted for the most common comorbid conditions in endometriosis (Pope et al., 2015).

In rodents, the EPM, the light-dark box and the open field test belong to the most used anxiety-related defense behavior assays (Mohammad et al., 2016). To our knowledge, there is only one study that has measured anxiety-related behaviors in EIM using the syngeneic transplantation model (Li et al., 2018). Li and colleagues have observed that endometriosis mice show a decreased time and distance traveled at 6, 8, 10, and 12 weeks after surgery compared to controls in the open field test, while locomotor function was unaffected. These observations demonstrate anxiety-like behavior in EIM and concur with our observations on a decreased time spent in the open arms of the EPM 5 weeks post-surgery without alterations in locomotive function. However, some differences in the methodology need to be highlighted: we performed two independent studies assessing anxiety at the same time post-surgery. In contrast, Li and colleagues measured anxiety in the same cohort of animals every 2 weeks post-surgery, which may lead to habituation and in consequence, a decrease in explorative behavior. Still, the distance traveled by both groups appeared stable along the time points measured. In our study, the percentage of time spent in the exposed region fully coincides with the average observed in a meta-analysis of preclinical models of anxiety, being 18% of total time in the exposed area a standard measure of anxiety (Mohammad et al., 2016).

Dysregulation of food consumption is another maladaptive response in anxiety disorders, being hypophagia, an increase in latency to begin feeding and decrease in the amount of ingested food, one trait in rodent models of anxiety that can be tested by assays like NEIFS (Cryan and Sweeney, 2011). In our observations, EIM tended to higher latencies to approach a palatable food compared to sham, which may argue for a mild anxiety-related behavior. However, this effect was maintained at day 5, when animals were back in their home cages, which may also argue for a generalized alteration of food motivation. Up to date we have not found other studies assessing NEIFS in endometriosis research. Since pharmacological administration of anxiolytics successfully reduces the latency to approach palatable food in anxiety-induced mice (Rex et al., 1996), it would be interesting to see if anxiolytic treatment would reduce these behaviors in EIM.

It is unclear which mechanisms may explain the occurrence of anxiety-related behavior in EIM. Sustained local inflammation in the endometriotic lesions and/or peritoneal cavity as described for women suffering from endometriosis is discussed as main driver for activation of peripheral nerve endings leading to chronic pelvic pain (Laux-Biehlmann et al., 2015b; McKinnon et al., 2015). Additionally, in the current ICD framework, endometriosis is characterized as “chronic visceral pain from persistent inflammation in the pelvic region” (Aziz et al., 2019), and low-grade chronic inflammation has been attributed to anxiety and depression in patients (Leonard, 2018). Kalfas et al. (2022), described a strong association between anxiety and pelvic pain in endometriosis patients. While we were not able to show generalized pain, it cannot be ruled out that EIM suffered from low chronic pelvic pain. It was shown recently, that induction of endometriosis in a syngeneic inoculation model leads to chronic abdominal pain (Greaves et al., 2017). The observed effects were associated with an over-representation of the PGE2 signaling pathway and nociceptive ion channels in endometriosis lesions of the animals suggesting ongoing inflammation. In context of other pain models it was shown that chronic pain conditions lead to anxiety-like behavior in rodents quantified in EPM (Caspani et al., 2014). Taken together, our model might connect both lines of evidence, showing consequently that sustained inflammation in the mouse endometriosis model associates with increased anxiety and might provide an approach with relevant face validity for this endometriosis-related co-morbidity.

On molecular level, both suture and inoculation models of endometriosis induced a pro-inflammatory state with increased concentrations of pro-inflammatory cytokines IL-1β and/or TNF-α (Cao et al., 2004; Zhou et al., 2010; Laux-Biehlmann et al., 2015a; Boyken et al., 2016; Mishra et al., 2020). These inflammatory processes might be the driver of the observed anxiety-related behavior, given that the pro-inflammatory cytokine IL-1β has demonstrated anxiogenic effects in mice (Munshi et al., 2019), and inflammatory processes including upregulation of IL-1b was described in peritoneal fluid of endometriosis patients (Mori et al., 1991). Another possibility is that endometriosis-pain chronically activate the hypothalamus-pituitary-adrenal (HPA) axis, leading to permanently increased cortisol levels and anxiety, as observed in endometriosis patients (Masciullo et al., 2021).

Interestingly, our results indicate the existence of anxiety-related behaviors in EIM without observing differences on generalized thermal- nor mechanical-pain. Other *in vivo* studies in surgically induced syngeneic models of endometriosis have observed generalized hyperalgesia, determined by decreases in hind paw withdrawal latencies to heat stimulation (Guo et al., 2013, 2015; Long et al., 2016, 2019; Ding et al., 2018) or thresholds to mechanical stimulation (Greaves et al., 2017), contrasting our observations. One possibility for this discrepancy is that the surgery was too invasive to distinguish pain thresholds between groups, lowering MPT in sham mice as well, and masking the pain-related behavior from the EIM. Another possibility is that EIM only develop local hyperalgesia in the abdominal/pelvic area. In this study, abdominal thresholds were only measured at the end of the experiment, and no differences were observed between groups (data not shown). We do not exclude that other type of endometriosis models would develop generalized hyperalgesia in EIM mice, or longer lasting local hyperalgesia in the abdominal area.

Others have observed local abdominal hyperalgesia to noxious mechanical (Greaves et al., 2017) or chemical (Tian et al., 2018) stimulation in preclinical models of endometriosis that can be attenuated following treatment (Guo et al., 2015; Greaves et al., 2017; Ding et al., 2018; Tian et al., 2018; Long et al., 2019). For example, Greaves et al. (2017) observed abdominal mechanical hyperalgesia in a syngeneic inoculation model 21–22 days post-inoculation. Authors performed an intraperitoneal injection of tissue derived from decidualized donor mice into an ovariectomized recipient mice supplemented with estradiol. Our study used surgical sutures of uterine horn biopsies into the mesentery and abdominal wall in normal cycling mice.

Measures of spontaneous, observer-independent pain behaviors are recommended to complement evoked-pain behaviors (Tappe-Theodor and Kuner, 2014). Although we did not observe differences on locomotor activity in the time points tested, it would be interesting to measure this in a longitudinal study. Furthermore, analysis of ultrasonic vocalizations or continuous monitoring of rodents’ behavior in their home cages have emerged as attractive alternatives to reveal the affective/emotional state of rodents as well as behaviors otherwise impossible to observe and have to potential to increase reproducibility and internal validity in translational research.

In summary, in a mouse model of endometriosis we observed increased anxiety-like behaviors, suggesting that the presence of endometriotic lesions in the abdominal cavity could, similarly to patients, induce psychopathological impairments in mice behavior. These endpoints might support identification of mechanisms relevant for the development of endometriosis-related comorbidities.

## Data availability statement

The raw data supporting the conclusions of this article will be made available by the authors, without undue reservation.

## Ethics statement

The animal study was reviewed and approved by American Preclinical Services Institutional Animal Care and Use Committee (IACUC).

## Author contributions

JN and TZ contributed to the conceptual design of experiments and data analysis. MH and OB contributed to the execution of the experiments. AL-B and PN-B contributed to data analyses and creation of figures. All authors contributed to manuscript writing and revision and approved the submitted version.
