# Supplementary material for: Anxiety-related behaviors without observation of generalized pain in a mouse model of endometriosis
Source: Front Behav Neurosci. 2023 Feb 9;17:1118598. doi: 10.3389/fnbeh.2023.1118598 (PMC9947402; doi:10.3389/fnbeh.2023.1118598)
Supplement: Supplementary file 1 [file Data_Sheet_1.docx]

# Supplementary Material
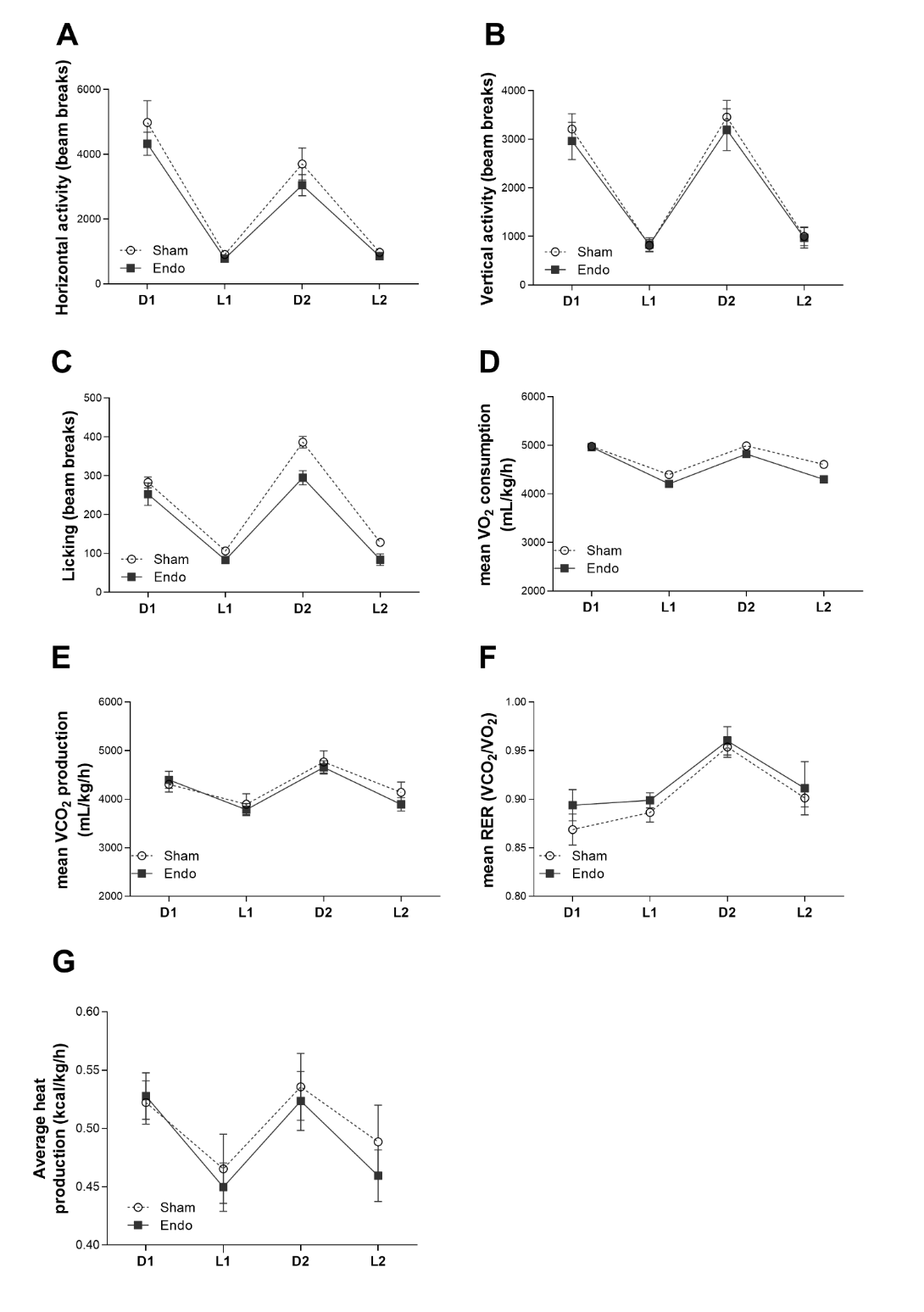


**Supplementary Figure 1: Surgical induction of endometriosis in mice does not alter locomotor activity nor metabolic parameters.** The CCMS assay was performed in EIM and sham mice 61-74 days after induction of endometriosis during 2 consecutive days. **A** and **B**: mean horizontal and mean vertical activity of EIM and sham mice. **C**: mean number of lickings, indicative of water consumption in EIM and sham mice. **D, E** and **F**: mean oxygen consumption, CO2 production and mean respiratory exchange ratio of EIM and sham mice. **G**: average heat production in EIM and sham mice. D1: Dark phase of day 1, L1: light phase of day 1. D2: dark phase of day 2. L2: light phase of day 2.
